# The Development of Adultoid Reproductives and Brachypterous Neotenic Reproductives From the Last Instar Nymphs in *Reticulitermes labralis* (Isoptera: Rhinotermitidae): A Comparative Study

**DOI:** 10.1093/jisesa/iev130

**Published:** 2015-10-22

**Authors:** Xiao Hong Su, Wei Xue, He Liu, Jiao Ling Chen, Xiao Jing Zhang, Lian Xi Xing, Ming Hua Liu

**Affiliations:** Biology Department, College of Life Sciences, Northwest University, Xi’an, China

**Keywords:** termite, secondary reproductive, juvenile hormone, vitellogenin gene expression, quantitative PCR

## Abstract

Secondary reproductives develop primarily from nymphs. However, they have been rarely studied; in particular, the development of adultoid reproductives (AR) with floppy wings is still unclear. In this study, the change in juvenile hormone (JH) levels, vitellogenin gene expression, and oogenesis during the development of AR and brachypterous neotenic reproductives (BN) from the last instar nymphs of *Reticulitermes labralis* are investigated and compared. The results showed that the AR derived from the last instar nymphs by molting, and they were more similar to neotenic reproductives in morphology. In addition, the paired AR were not able to survive in the absence of workers. In *R. labralis, *the process of the last instar nymphs developing into AR and BN took an increase in JH level as a starting point. The JH level of the last instar nymphs molting into BN was approximately 1.5-fold higher than that of the AR. Additionally, The JHIII level of BN peaked on day 5, and that of AR peaked on day 10, which induced the onset of vitellogenesis in BN and AR, respectively. After molting, the vitellogenin gene expression levels of both BN and AR initially increased and then declined, and the expression levels in the BN were significantly higher than those in the AR. In addition, the oocytes of BN matured earlier than those of the AR, and the number of eggs laid by the BN was higher than the number laid by the AR. Our results demonstrate that, in *R. labralis*, the last instar nymphs can develop into AR, which are significantly different from BN in their development.

Lower termites are characterized by a unique flexibility in development (Korb et al. 2009). Caste development in the genus *Reticulitermes* within Rhinotermitidae, as lower termites, is highly plastic, so that environmental stimuli can direct the developmental pathway (Elliott and Stay 2007, Korb and Hartfelder 2008). In *Reticulitermes*, newly hatched individuals (larvae) develop into workers or nymphs, which are distinguished by the absence or presence of wing pads, respectively. The primary reproductives develop via the last instar nymphs molting into winged sexuals (alate adults) that fly off at the same time in great swarms, mate, and create new colonies. In the absence of the primary reproductives, some of the workers and nymphs develop into secondary reproductives to continue colony growth. The workers become apterous neotenics, and the nymphs become brachypterous neotenics (Thorne et al. 1999, Korb and Hartfelder 2008). Roisin and Pasteels (1991) and Sieber (1985) reported that in Kalotermitidae and Termitidae, adultoid reproductives (AR) occurred as alate individuals who shed their wings and reproduce within the natal colony to become secondary reproductives. Neoh et al*.* (2010) suggested that the AR in *Macrotermes gilvus* (Termitidae) developed from alate adults with torn wings and retained at the original nest as secondary reproductives. However, in *Reticulitermes urbis *and *R. labralis*, the AR with floppy wings develop from nymphs and have the secondary reproductive function (Ghesini and Marini 2009, Xing et al. 2015). Field and laboratory colonies of *Reticulitermes* contain a large number of secondary reproductives derived from nymphs, in which the secondary reproductives play important roles in colony growth (Huang et al. 2013, Wu et al. 2013). Therefore, the study of differentiation of secondary reproductives from nymphs has important significance for understanding the expansion of the colony.

All the individuals in a termite colony share similar genetic backgrounds. The maintenance and transformation of the caste structure are controlled by environmental factors and pheromones that induce gene expression based on their endocrine signature (Korb and Hartfelder 2008; Korb et al. 2009, 2012). Juvenile hormone (JH) is a central regulator of termite postembryonic development and life history traits (Korb 2015). Nijhout and Wheeler (1982) proposed a model for caste differentiation of termites, in which continuous low JH titers would induce alate adult differentiation, whereas high JH titers followed by low titers would induce neotenic reproductive differentiation (Cornette et al. 2008). In *Reticulitermes flavipes*, the development of a worker into an apterous neotenic reproductive requires somewhat elevated JH synthesis immediately prior to the molt (Elliott and Stay 2008). The increase in JH titers can predict the differentiation of secondary reproductives in *Kalotermes flavicollis* (Nijhout and Wheeler 1982).

The reproductive maturity of female individuals is signified by the emergence of vitellogenic oocytes in insects. Vitellogenin has been considered a sex-specific protein that is exclusively required for oocyte vitellogenesis (Korb and Hartfelder 2008). During vitellogenesis, vitellogenin is synthesized in the fat body, secreted into the hemolymph and taken up by the developing oocyte. JH in hemolymph stimulates vitellogenin gene expression and promotes vitellogenesis of oocytes (Borst et al. 2000, Parthasarathy et al. 2010). For *Reticulitermes speratus* queens, the increased JH titers are positively correlated with high levels of vitellogenin gene expression (Maekawa et al. 2010). However, at different stages of oogenesis in *R. flavipes*, JH does not continuously stimulate vitellogenesis of the reproductives. For example, when the oocytes were at the early stage of vitellogenesis, the rate of JH synthesis was high; when the oocytes were at the stages before and after vitellogenesis, the rate of JH synthesis was low (Elliott and Stay 2007). In *Zootermopsis angusticollis*, an elevated JH titer prevents precocious ovarian activity in immature alates or stimulates oogenesis in mature queens. Therefore, JH plays a dual role in the reproductives depending on their developmental stage (Brent et al. 2005, Maekawa et al. 2010).

Although both AR and brachypterous neotenic reproductives (BN) from the last instar nymphs are observed in field and laboratory colonies of *Reticulitermes*, the developmental differences between them are still unclear. In particular, the development of AR has been rarely studied. To better understand the regulation mechanisms of the development of AR and BN and to elucidate the biological characteristics and reproductive functions of AR, in this study, the dynamic changes in JH levels, vitellogenin gene expression and oogenesis during the development of AR and BN of *R. labralis* from the last instar nymphs are investigated and compared for the first time.

## Materials and Methods

### 

#### Termites

The experimental termites (*R. labralis*) were collected at Daxingshang Temple, Xi'an City, China, in April 2012 when the alate adults were flying off the rotten wood, and a large number of AR were found in the colonies. Three mature colonies of *R. labralis* were brought back to the laboratory, and the alate adults and AR were collected immediately. The wood was placed on the ground in the orchard of Northwest University under a humid, shaded area, and covered with moist sand soil.

To confirm whether a pair of AR of *R. labralis* could create a new colony similar to the alate adults, AR and alate adult individuals from natal colonies were paired, one female to one male, and 50 pairs of AR and 50 pairs of alate adults were established, respectively. Sterilized pine tree sawdust that was wet with distilled water was compacted in a 30 ml transparent bottle in which the sawdust took up 4/5 of the volume in the bottle. Each AR or alate adult pair was placed in a bottle and kept at 25°C under constant darkness. The pairs were observed daily, and their mortality was recorded.

In August, the last instar nymphs (the sixth-instar nymphs) appeared in the field colonies. To obtain AR and BN that developed from the last instar nymph under laboratory conditions, some of the last instar nymphs and workers were isolated from their natal colonies. One female last instar nymph and 10 workers formed a group (NW group) and were placed in 30 ml transparent bottles packed with sawdust at 25°C; 500 replications from three mature colonies were established.

At 0 (time of molting), 5, 10, 15, and 20 d after the last instar nymphs in the NW groups molted to AR and BN and when eggs were found in the groups, the samples were collected, immersed immediately in liquid nitrogen, and stored at −80°C. Female last instar nymphs from the natal colonies and NW groups that were cultured for 5 d were also sampled and stored at −80°C for use in the JH and RNA extractions. For histological observations of the ovarian development of AR and BN, the samples were fixed using Bouin solution, stored at −4°C, and eventually stained with HE (hematoxylin and eosin).

#### Histological Observations of Ovarian Development

The degree of ovarian developments of the AR and BN was evaluated by counting the numbers of ovarioles. The oocyte development of the AR and BN was evaluated with HE staining. The fixed samples were dehydrated in an ascending ethanol series and embedded in paraffin. Longitudinal 7 μm sections were processed by a microtome and collected on polylysine-coated slides. The deparaffinized and rehydrated sections were stained with HE. The sections were observed using digital microscopes (Olympus Corporation, Japan), and the number of vitellogenic oocytes was counted in each individual to evaluate the level of ovarian maturation.

#### JH Extraction

Previously reported extraction methods with modifications were applied (Cornette et al. 2008, Maekawa et al. 2010). Briefly, the individuals that were stored at −80°C were homogenized in 2 ml of methanol/isooctane (1:1, v/v), and the homogenate was allowed to stand at room temperature for 30 min. After centrifugation at 13,900 × *g* for 5 min, the supernatant was collected and mixed with 10 µg of fenoxycarb (Sigma, St Louis, MO, USA) as an internal standard. The mixture was vortexed and allowed to stand at room temperature for 30 min before centrifugation at 5,600 × *g* for 15 min. The upper isooctane phase was transferred into a new glass vial, and the methanol phase was vortexed and centrifuged at 7,700 × *g* for 30 min and then combined with the isooctane phase. The resulting mixture was dried under a stream of nitrogen, and the dried pellets were dissolved in 20 µl of methanol.

#### Liquid Chromatography-Mass Spectrometry

The JH levels in the samples were analyzed using liquid chromatography-mass spectrometry (LC-MS) according to Maekawa et al. (2010). From each 20 μl concentrated sample, 5 μl was separated on a 150 by 2-mm-inner-diameter C18 reverse-phase column (Shimadzu, Science Inc., Kyoto, Japan) protected by a guard column (Scienhome Scientific Inc., Tianjing, China) with a gradient elution of water/methanol (80–100% methanol over 0–15 min and 100% methanol for 5 min) at a flow rate of 0.2 ml/min, utilizing an Agilent 1100 high-performance liquid chromatography system with an autosampler (Agilent Technologies, Santa Clara, CA). A mass spectral analysis was performed using electrospray ionization in the positive mode of a micro TOF-HS (Bruker Daltonik GmbH, Bremen, Germany) under the following conditions: electrospray capillary set at 4.5 kV and dry temperature of 200°C. The nitrogen pressure for the nebulizer was 1.6 bar, and the drying gas nitrogen flow rate was 9 liters min^− 1^. Quantification of JH III and fenoxycarb was performed by monitoring the [M + H]^+ ^and [M+ Na]^+ ^ions. A calibration curve for JH III (Sigma, St Louis, MO) was plotted using the same internal standard concentration as fenoxycarb in each sample. The JH III level from each sample was then calculated after analysis of the chromatogram data using quantification software (Bruker Daltonics, Bremen, Germany). Data are expressed as ng mg^−1^ fresh weight.

#### cDNA Preparation and Real-Time Quantitative Polymerase Chain Reaction

Total RNA was extracted from individual termites (removed intestines) and stored at −80°C using a FastPure RNA kit (Takara, Bio. Inc., Tokyo, Japan). Three different individuals were used for each category. After the DNase (Takara, Bio. Inc., Tokyo, Japan) treatment, the quality and quantity of the extracted RNA were determined by spectroscopic measurements at 230, 260, and 280 nm using a NanoVue spectrophotometer. cDNA was synthesized from 50 ng mRNA(DNase-treated) with an oligo-dT15 primer and Superscript II reverse transcriptase (Takara, Bio. Inc., Tokyo, Japan) and then incubated at room temperature for 10 min and 37°C for 30 min (Scharf et al. 2005, Shimada and Maekawa 2010).

Quantitative polymerase chain reaction was performed as previously described (Maekawa et al. 2010, Shimada and Maekawa 2010) with beta-actin (forward: AGC GGG AAA TCG TGC GTG AC, reverse: CAA TGG TGATGA CCT GGC CAT) as a control gene because it was evaluated as the most reliable reference gene in *R. flavipes* (Scharf et al. 2005, Zhou et al. 2006). Primers for the vitellogenin | (Vg |) gene were designed by Invitrogen Trading (Shanghai) Co., Ltd. based on the nucleotide sequence of the Vg | gene of *R. flavipes* (accession number BQ788169, forward: CCT ACA TGC GTT GAT GG; reverse: TGA CGA CTA TGC ACT CCA GC). The relative quantification of the cDNA was performed using a SYBR Green I chemistry and LightCycler 480 System detection system (Roche Diagnostics, Basel, Switzerland).

## Results

### 

#### The Paired AR Were Not Able to Found New Colonies

Four groups of the female–male pairs of AR died after 1 d, and all the 50 pairs died within 2 wk. Tunnel digging behavior was not observed in the paired AR. The paired dealate adults started to dig tunnels in 3–7 d, and downward tunnels were clearly visible. After 2 wk, 44 pairs survived and only six pairs died. The paired alate adults laid eggs after 3 wk.

#### The Differentiation and Morphology of AR and BN

**Fig. 1.**
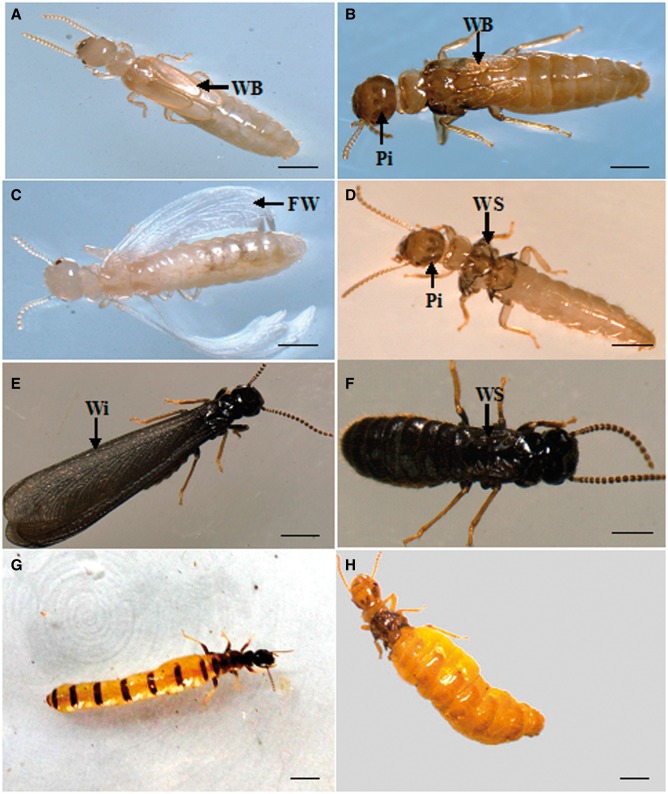
The morphological differences among the last instar nymphs, brachypterous neotenics, AR, and alate adults in *R. labralis*. (A) The last instar nymphs had a milky white body and long wing buds (WB). (B) The brachypterous neotenics had dark brown base at the wing buds (WB), a light brown body and pigmentation of dark brown stripes on the head (Pi). (C) The AR had floppy wings (FW) and a light brown body. (D) The AR after dealation, with wing scales (WS) and pigmentation of dark brown stripes on the head (P). (E) The alate adults with darkened pigmentation, higher levels of sclerotization, and black body and wings (Wi). (F) The alate adults after dealation with wing scales (WS). (G) The primary reproductive as queen from the field colony. (H) The adultoid reproductive as queen from the field colony. Scale bar = 1.0 mm.

Last instar nymphs in the NW groups of *R. labralis* started molting after 1 wk, and 67.5% of last instar nymphs developed into AR and 32.5% developed into BN via a single molt in 9 wk. There were apparent morphological differences among the last instar nymph, AR, BN, and alate adult individuals. The last instar nymphs had a milky white body, long wing buds, and a lack of dark brown stripes on the head (Fig. 1A). The BN had long wing buds too, but a light brown body, extended intersegmental membranes, dark brown stripes on the head, and dark brown buds (Fig. 1B). The body color of the AR was milky white after molting and then turned into light brown. The AR had floppy wings which were unsclerotized and incapable of flight (Fig. 1C). These nonfunctioning wings were shed in 1 wk and left two pairs of wing scales at the mesonotum and metanotum. The AR also had dark brown stripes on head (Fig. 1D). Compared with the alate adult individuals (Fig. 1E and F), the AR and BN had lower levels of sclerotization and lighter pigmentation. The last instar nymphs could develop into AR and BN whenever a few the last instar nymphs with workers were isolated from their natal colonies, whereas alate adults from last instar nymphs were produced only in the nuptial flight period (in April).

#### The JH Levels During the Differentiation and Development of AR and BN

**Fig. 2.**
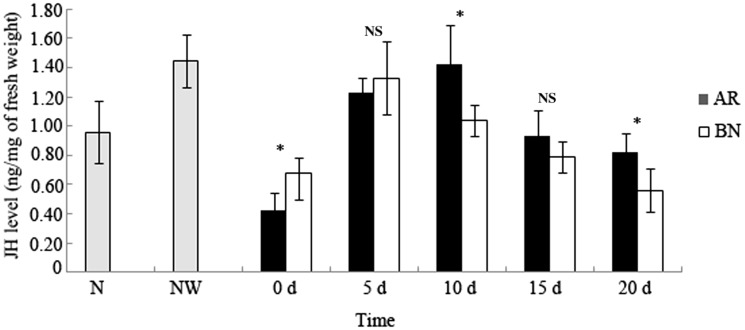
Changes in JHIII levels in *R. labralis* during development of the female AR and female BN from the last instar nymphs (*n* = 5). *N*, the female last instar nymphs from the field colony; NW, the isolated group consisting of one female last instar nymph and ten workers. The columns represent the means, and the bars represent the SD. *Significantly different (*P* < 0.05); NS: not significant (Welch’s *t*-test).

The JHIII level of the female last instar nymphs in the natal colonies was 0.96 ± 0.21 ng/mg. The JHIII level of the female last instar nymphs in the NW groups rapidly increased to 1.45 ± 0.18 ng/mg in the fifth day of culture (Fig. 2). After 1 wk when the last instar nymphs were molting to AR and BN, the JHIII level decreased to 0.42 ± 0.12 ng/mg and 0.69 ± 0.18 ng/mg, respectively. After AR and BN differentiation, their JHIII levels significantly increased. For AR, the JH level reached 1.23 ± 0.10 ng/mg 5 d after molting. Subsequently, the increasing trend slowed and peaked at 1.42 ± 0.32 ng/mg 10 d after molting and decreased to 0.93 ± 0.18 ng/mg and 0.82 ± 0.13 ng/mg, respectively, 15 d and 20 d after molting. The JHIII level of BN peaked at 1.31 ± 0.24 ng/mg 5 d after molt and decreased thereafter to 0.56 ± 0.15 ng/mg 20 d after molting. Within 20 d after the molt, the JHIII levels of both AR and BN underwent a dynamic change of an initial increase and then a decline.

The JHIII level during last instar nymph molting to BN was approximately 1.5-fold higher compared with that during the last instar nymph molting to AR. Although there was no significant difference between the maximum JHIII levels of AR and BN during their developmental processes, the JHIII level of BN peaked on day 5, whereas that of AR peaked on day 10 and started to decrease thereafter. Therefore, the dynamic change in the JHIII level in the BN was faster than that in the AR (Fig. 2).

#### Vitellogenin Gene Expression in AR and BN

**Fig. 3.**
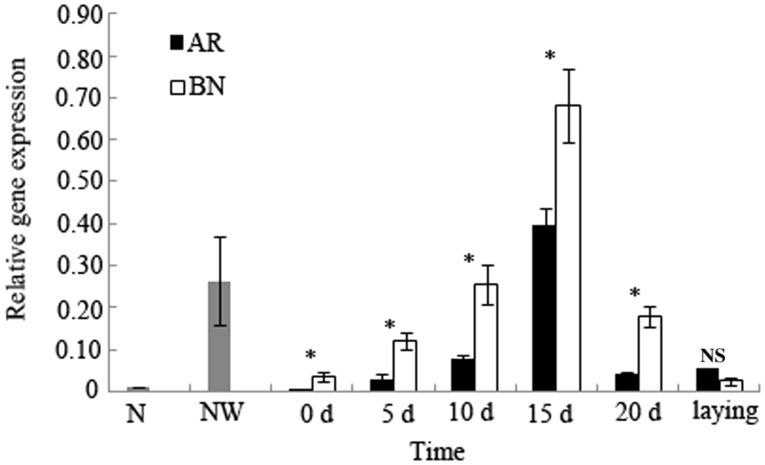
The relative expression levels of the vitellogenin gene in *R. labralis* during development of the AR and the BN from the last instar nymphs (*n* = 5). *N*, the female last instar nymphs from the field colony; NW, the isolated group consisting of one female last instar nymph and ten workers. The columns represent the means, and the bars represent the SD. *P < 0.05; NS, not significant (Welch’s *t*-test).

The relative expression level of the vitellogenin gene in the female last instar nymphs from the field colonies was 0.0102 ± 0.0025, whereas that from the NW groups for 5 d rapidly increased to 0.2640 ± 0.1042, which was approximately 26-fold higher compared with that of the field colonies (Fig. 3). One week later when the last instar nymph were molting into AR and BN, the relative expression levels of the vitellogenin gene decreased to minimum values of 0.0036 ± 0.0015 and 0.0358 ± 0.0129, respectively, with the relative expression levels of the BN approximately 10-fold higher compared with that of the AR.

The relative expression level of the vitellogenin gene in the AR increased rapidly 10 d after molting, peaking at 0.3956 ± 0.0787. The BN and AR began to lay eggs on day 22 and day 24, respectively. The relative expression level of the vitellogenin gene after egg laying was 0.0556 ± 0.0082, a level close to that on day 10 after molting. The relative expression level of the vitellogenin gene in the BN increased rapidly over the period from days 0 to 15 after molting and reached its peak value of 0.6811 ± 0.1215 on day 15, which was approximately 2-fold higher compared with that of the AR. After egg laying, the relative expression level of the vitellogenin gene in the BN reached its lowest point of 0.0293 ± 0.0074. The relative expression levels of the vitellogenin gene in the BN during the differentiation and maturation were significantly higher than that in the AR (Fig. 3).

#### Ovarian Development and Oogenesis of AR and BN

**Fig. 4.**
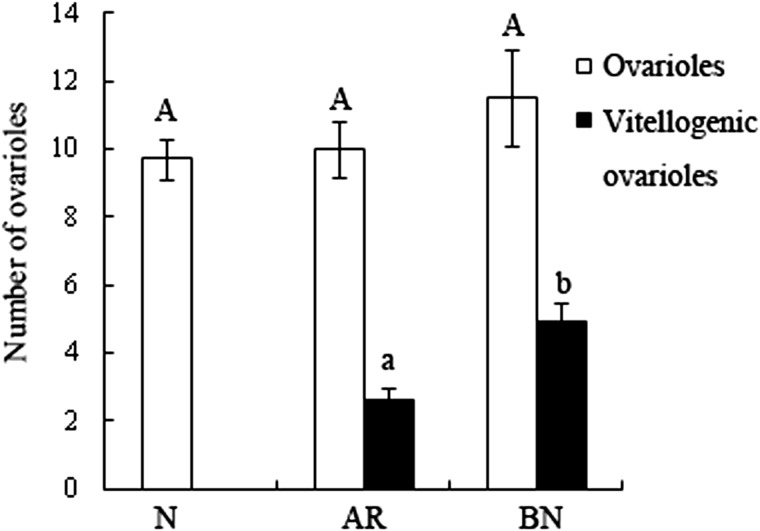
The mean number of ovarioles per ovary in the last instar nymphs (*N*), AR and BN of *R. labralis* (*n* = 5)*.* The columns represent the means, and the bars represent the SD. Letters (a and b) above the columns of the same color indicate significant differences (*P* < 0.05) between the AR and BN.

The mean number of ovarioles per ovary did not change significantly from last instar nymphs into AR and BN. Although there was no significant difference in the number of ovarioles between the AR and BN, the physogastric BN had more vitellogenic ovarioles compared with the physogastric AR (Fig. 4).

**Fig. 5.**
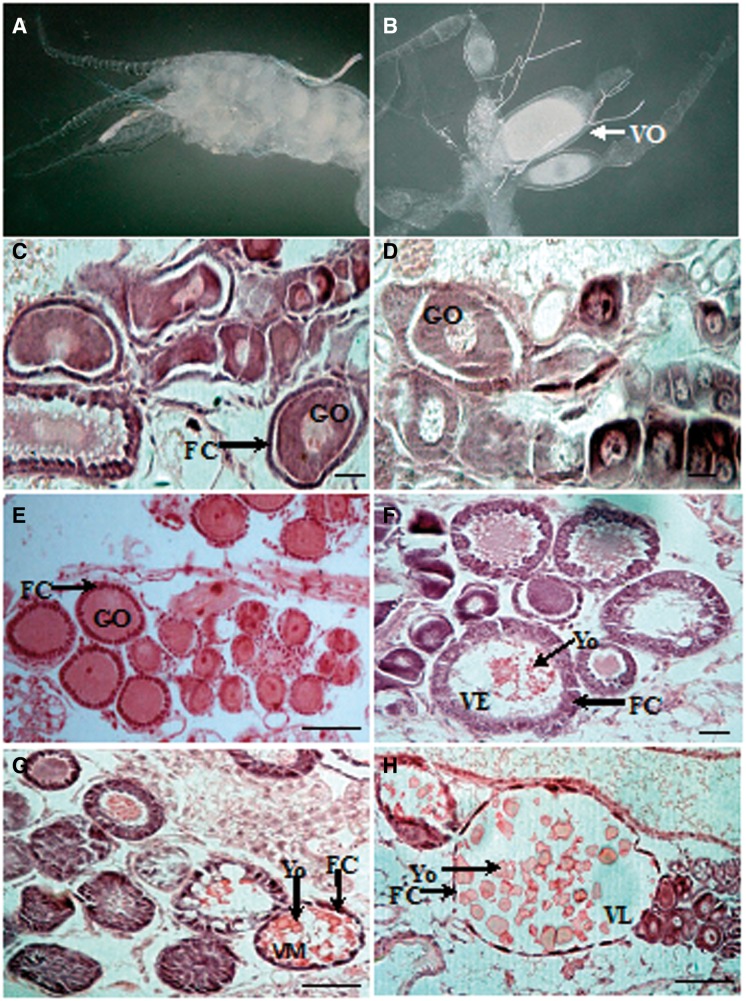
Oogenesis during the development of AR and BN from the last instar nymphs of *R. labralis.* (A) The ovary of the last instar nymph. (B) The vitellogenic oocytes (VO) in the ovary of adultoid reproductive. (C) At stage II of oogenesis of the last instar nymphs in natural colonies, each oocyte (GO) was surrounded by a layer of follicular cells (FC). (D) During the molting and differentiation process from the last instar nymphs to AR and BN, the development of oocytes was in stage II, and the regional thickening of the follicle cell layer (FC) was visible. (E) On day 10 of adultoid reproductive development, the development of oocytes was in stage II. (F) On day 15 of adultoid reproductive development, oogenesis was at the stage of early vitellogenesis (VE) with visible vitellogenic oocytes. (G) On day 20 of adultoid reproductive development, oogenesis was at the stage of mid-vitellogenesis (VM). (H) The BN on day 20, the oocytes at the late vitellogenesis stage (VL) were surrounded by elongated follicle cells (FC). (Yo) yolk. Scale bar = 15 µm.

The oogenesis of *Reticulitermes* includes three stages: oogonium differentiation stage (stage I), oocyte growth stage (stage II), and oocyte vitellogenesis stage (stage III) (Su et al. 2014). In *R. labralis, *the process of oogenesis in the last instar nymphs reached stage II (Fig. 5A and C). After 1 wk, during the last instar nymphs molting to AR and BN, the development of oocytes remained at stage II (Fig. 5D). On day 10, vitellogenic oocytes were not observed in the ovaries of AR (Fig. 5E). On day 15, vitellogenic oocytes were observed in the ovaries of AR, and they were at the early vitellogenesis stage with cylindrical follicle cells (Fig. 5F). On day 20, the development of oocytes of AR was at the mid-vitellogenesis stage (Fig. 5G). On day 10 of BN development, there were vitellogenic oocytes in the ovarioles, and on day 20, the oocyte development reached the late vitellogenesis stage when the size of the oocytes was the largest, and each oocyte was surrounded by a thin follicle cell layer, indicating that the follicle cells had begun to degenerate, and the oocytes had matured (Fig. 5H). Thus, compared with those of the AR, the oocytes of the BN matured earlier.

On day 10, the mean number of vitellogenic oocytes of the BN and AR was 3 ± 0.7 and 0, respectively (*n* = 5); on day 15, the mean number increased to 3.3 ± 1.6 and 1.8 ± 0.3, respectively (*n* = 5), and on day 20, it reached 4.3 ± 0.6 and 3.3 ± 0.5, respectively (*n* = 5). The BN and AR began to lay eggs on day 22 and day 24, respectively; the mean number of BN and AR eggs was 2.7 ± 0.6 and 1.3 ± 0.5 (*n* = 5), respectively.

## Discussion

This study demonstrated that in *R. labralis*, AR were derived from last instar nymphs, and the AR would thus fall into the category of secondary reproductives. The reproductives with light brown pigmentation and unsclerotized wings have been reported in *R. urbis *by Ghesini and Marini (2009)*. *However, Neoh et al. (2010) suggested that the AR of *M. gilvus* (Termitidae) originated from alate adults with damaged wings and stayed in the natal colonies to become secondary reproductives; they were morphologically indistinguishable from the primary reproductives. The presence of wings might lead to the conclusion that the AR of *R. labralis* are primary reproductives (alate adults), but their overall appearance is more similar to that of secondary reproductives (neotenic reproductives), and their wings are not functional. In *R. labralis*, it was observed that there were significant differences in level of exoskeleton sclerotization between the AR and alate adults. The insect exoskeleton is sclerotized. The exoskeleton as their major system for support, protection, and movement has profound effects on specific aspects of their method of growth, physiology, and muscle attachment (Romoser and Stoffolano 1999). Therefore, the lower level of sclerotization in the AR of *R. labralis* directly affects wing movements. In addition, the results of this study showed that the AR of *R. labralis* could develop from the last instar nymphs regardless of “nuptial flight season,” and they were not able to survive in the absence of workers; in contrast, last instar nymphs were only able to develop into alate adults in the nuptial flight season, and paired alate adults created new colonies even if their wings were damaged or were manually removed. Obviously, the AR are markedly different from the alate adults; except for having wings, they are more similar to the neotenic reproductives in morphology and behavior.

Not all species of termites produce AR. On studying *Reticulitermes aculabialis* for many years, we did not observe AR; the AR is rarely reported in other *Reticulitermes* species (Ghesini and Marini 2009). Therefore, the AR are not common in the genus *Reticulitermes. *In this study, the majority of the isolated last instar nymphs from the natal colonies of *R. labralis* developed into AR, whereas only a small portion developed into BN. However, in field colonies, almost all the last instar nymphs of *R. labralis* do not develop into reproductives until their nuptial flight season (April) when the majority of the last instar nymphs become alate adults and disperse, and a small portion develop into AR remaining in the natal colony. Therefore, the early separation from the field colony may have accelerated last instar nymphs development towards sexual maturity and prevented their development into normal alate adults. The differentiation of AR in *R. labralis* expanded the developmental pathways of the last instar nymphs, making the mating system and life cycle of *Reticulitermes* more complex. We suggest that AR is a middle reproductive caste between the BN and the alate adults, which implicates evolutionary history of reproductive type in *Reticulitermes.*

In *R. labralis, *the process of the last instar nymphs developing into secondary reproductives takes an increase in JH level as a starting point, and compared with developing into AR, the last instar nymphs developing into brachypterous neotenics require higher JH levels*,* indicating that the level of JH influences the conversion direction of last instar nymphs in *R. labralis*. The last instar nymphs required dramatically increased JH levels before molting to reproductives. However, when the last instar nymphs of *R. labralis* were molting to AR and brachypterous neotenics, their JH levels were maintained at very low levels. The low JH titer during molting might result from the influence of the ecdysteroids because ecdysteroids may inhibit corpora allata (CA) activity by promoting the release of allatostatins or by causing variations in the sensitivity of the CA to the presence of the neuropeptides (Brent et al. 2005). The JH levels in the last instar nymphs that were molting into brachypterous neotenics is approximately 1.5-fold higher than that of molting into AR. This suggests that the differentiation of brachypterous neotenics appears to require higher JH level than is required for AR. The differentiation of individuals is associated with JH regulation; for example, in *R. flavipes*, the workers that were destined to become pre-soldiers have 2.5-fold higher JH synthesis than the workers that would develop into neotenic reproductives (Elliott and Stay 2008).

The appearance of the vitellogenic oocytes in the brachypterous neotenics was earlier than that in the AR, which was associated with the JH titer reaching its maximum value earlier in the brachypterous neotenics. In *R. labralis*, the JH levels of the brachypterous neotenics and AR reached their maximum values on days 5 and 10 after molting, respectively, whereas their vitellogenic oocytes occurred on days 10 and 15, respectively. Therefore, it is believed that the peak level of JH induces the initiation of oocyte vitellogenesis in *R. labralis*. In Orthoptera, the concentration of JH has been shown to be the critical factor that determines the onset and continuation of vitellogenesis (Borst et al. 2000). Previous studies have reported that in the first cycle of egg development in *R. flavipes*, CA with high rates of JH synthesis were from females with early vitellogenic oocytes, whereas CA with low rates of JH synthesis were from females with either pre-vitellogenic or mature oocytes (Elliott and Stay 2007), which indicated the elevated JH level preceded vitellogenesis and high JH titer-induced vitellogenesis and maturation of the oocytes (Maekawa et al. 2010, Song et al*.* 2014). Each oocytes of *R. labralis* are surrounded by a layer of follicle cells. It has been shown that JH regulates the uptake of vitellogenin by interacting with membrane receptors on the follicle cells in *Locusta migratoria* (Davey et al. 1993). JH stimulates the follicular cells and leads to the formation of channels between the adjacent follicle cells for the intake of yolk, and it provides stimulation to enlarge the follicular cells and increase the DNA synthesis rate (Koeppe et al. 1980, Clifton and Noriega 2012). In addition, this study showed that in *R. labralis*, the JH level of the reproductives with the developing oocytes was higher than that of the last instar nymphs from natal colonies; however, the JH level of the reproductives with oocytes at late vitellogenesis was lower than that of the last instar nymphs from natal colonies. This result is most likely due to the stimulation of JH synthesis by developing oocytes, and the inhibition of JH synthesis by almost mature oocytes.

Interestingly, although the vitellogenin gene expression levels of both brachypterous neotenics and AR of *R. labralis* initially increased and then declined, the vitellogenin gene expression level of the brachypterous neotenics with lower JH levels was significantly higher than that of the AR with higher JH levels, indicating that the vitellogenin gene expression in brachypterous neotenics is very sensitive to JH induction. In addition, the JH levels of the brachypterous neotenics and AR peaked on days 5 and 10 after molting, respectively. Obviously, in the brachypterous neotenics, higher levels of the vitellogenin gene expression may be related to a faster peak in the JH level. Generally, in insects, the vitellogenin gene expression level is regulated by JH, which directly stimulates the transcription of vitellogenin genes in the fat body (Borst et al. 2000, Maekawa et al. 2010, Parthasarathy et al. 2010). The vitellogenin gene expression level also affects oogenesis and the number of vitellogenic oocytes in individuals. Compared with AR, the brachypterous neotenics lay more eggs and lay the eggs earlier, indicating that the brachypterous neotenics have a relatively high reproductive capacity.
